# Supplementary material for: A Systematic Review of Uremic Toxin Concentrations and Cardiovascular Risk Markers in Pediatric Chronic Kidney Disease
Source: Toxins (Basel). 2024 Aug 8;16(8):345. doi: 10.3390/toxins16080345 (PMC11358976; doi:10.3390/toxins16080345)
Supplement: Supplementary file 1 [file toxins-16-00345-s001.zip › toxins-3049267-supplementary.pdf]

Supplementary Materials

Table S1. Results of eligible articles.

Study	Aim(s)	Outcome Parameters	Nephro-cardiac Interplay	Uremic Toxin Changes	Cardiac Changes
Crespo-Salgado et al., 2016 [19]	- To assess the impact of ESKD on the intestinal microbiota of pediatric patients.<br>- To evaluate how changes in the microbiome could influence the production and serum levels of bacterially derived uremic toxins.<br>- To investigate the correlation between microbiota composition, C-reactive protein (an inflammation marker), and D-lactate (indirect marker of intestinal permeability).	- Relative abundance of bacterial taxa and diversity within the gut microbiome<br>- Serum levels of bacterially derived uremic toxins<br>- Markers of systemic inflammation and intestinal permeability	- The paper does not directly address the interplay between nephrological and cardiac disorders in pediatric CRS.	- Serum levels of bacterially derived uremic toxins, IS and pCS, were significantly increased in pediatric patients with ESKD.<br>- IS and pCS levels were significantly higher in patients undergoing PD and HD compared to the healthy controls and kidney transplant recipients.<br>- There were no significant differences in IS and pCS levels between PD and HD patients.<br>- The altered intestinal microbiota in these ESKD patients, characterized by bacterial population shifts and decreased bacterial diversity, was associated with increased production of these uremic toxins.	- Although the primary focus of the paper was not on direct cardiac changes, the study implied a connection between increased uremic toxins and inflammation, which is a known contributor of CVD in ESKD patients.
Holle et al., 2019 [20]	- To investigate the association of uremic toxins (IS and pCS) with CV	- cIMT<br>- PWV<br>- LVMI	- The paper does not directly address the interplay between nephrological and cardiac disorders in pediatric CRS.	- Serum levels of IS and pCS showed significant positive associations with urea levels	- Only IS was significantly associated with a higher baseline cIMT and progression of PWV over 12

	surrogate parameters in children with CKD.		- However, the findings link renal dysfunction (through the accumulation of uremic toxins due to reduced renal clearance) with CV alterations.	and negative associations with eGFR and uric acid.<br>- These associations indicated that as kidney function deteriorated, the levels of these toxins increased.	months, both surrogate markers of CV health.
Holle et al., 2020 [21]	- To assess the association of serum IS concentrations with the progression of CKD in children, specifically the potential predictive value of IS for disease progression.	- Progression of CKD, defined by a 50% loss of eGFR, eGFR < 10 ml/min/1.73m², or the initiation of renal replacement therapy.	- The paper does not directly address the interplay between nephrological and cardiac disorders in pediatric CRS.<br>- It discussed the relationship between uremic toxins and kidney disease progression.	- Serum IS levels, but not serum pCS levels, were significantly associated with faster CKD progression, independent of other traditional risk factors such as baseline eGFR, proteinuria, and blood pressure.	- No findings about specific cardiac changes or the direct impact of uremic toxins on cardiac function.
Özdemir et al., 2015 [22]	- To investigate the functional and structural CV system changes in children receiving dialysis.<br>- To explore any associations between these CV changes and the clearance of small water-soluble, middle molecule, and protein-bound uremic toxins.	- Clearance of uremic toxins in blood samples from HD patients with or without CVD<br>- AIx<br>- PWV<br>- cIMT<br>- LVMI	- The study does not explicitly mention CRS, but it examines the relationship between uremic toxin clearance and CV changes.	- There was no significant difference in the clearance of small water-soluble uremic toxins between patients with and without cardiac dysfunction.<br>- The clearance of β2M from the class of middle molecule uremic toxins and homocysteine from the class of protein-bound uremic toxins were significantly low in HD patients with CVD.<br>- Between the two dialysis modalities, middle molecule	- Mean values of AIx, PWV, cIMT, and LVMI were significantly worse in the 40% of HD patients and 20% of PD patients compared to the control group.<br>- Lower clearance of middle molecule and protein-bound uremic toxin classes was associated with worse CV outcomes in pediatric patients on HD.

				and protein-bound uremic toxins were more efficiently cleared by PD compared to HD.	
Snauwaert et al., 2018 [23]	- To describe and compare the concentrations of various uremic toxins in healthy children and those with non-dialysis CKD stages 1–5.<br>- To establish reference values for these toxins in the pediatric population.	- Serum concentrations of small water-soluble, middle molecule, and protein-bound uremic toxins	- The paper does not directly address the interplay between nephrological and cardiac disorders or reference pediatric CRS.<br>- It discussed the analysis of uremic toxin levels in children with various stages of CKD compared to healthy controls.	- Established reference values for a range of uremic toxins belonging to each class in healthy children and in children with non-dialysis CKD.<br>- Concentrations of SDMA, CfD, β2M, HA, IAA, IS, pCS, and CMPF were significantly higher in the overall CKD group compared to healthy controls.<br>- Concentrations of SDMA, CfD, β2M, IS, and CMPF were significantly higher in CKD stages 1–2 onwards compared to healthy controls.<br>- Concentrations of pCS, pCG and IAA were significantly higher in CKD stages 3-4 onwards compared to healthy controls.<br>- Concentrations of ADMA and HA were significantly higher only in CKD stage 5	- No findings about specific cardiac changes or the direct impact of uremic toxins on cardiac function.

				compared to healthy controls.	
Snauwaert et al., 2019 [24]	- To investigate how RKF influences the concentrations of various uremic toxins in pediatric patients undergoing maintenance HD.	- Levels of protein-bound uremic toxins<br>- Percentage of protein binding of these toxins<br>- Residual urine volume	- The paper does not directly address the interplay between nephrological and cardiac disorders or reference pediatric CRS.<br>- It discussed the relationship between uremic toxin concentrations and RKF in pediatric HD patients.	- 7.8-fold rise in IAA and 43-fold rise in HA in children on HD compared to healthy controls.<br>- Levels of β2M, pCG, HA, IAA, and IS were significantly increased in children on HD compared to children with non-dialysis CKD stages 4-5.<br>- There were no significant differences in UA, pCS, CMPF levels between children on HD and children with non-dialysis CKD stages 4-5.<br>- The degree of protein-binding of all protein-bound uremic toxins was significantly less in children on HD compared to children with non-dialysis CKD stages 4-5.<br>- Increased total levels of pCG, HA, IS, and pCS showed significant positive associations with age in children on HD<br>- β2M, HA, and CMPF levels were significantly higher in children on anuric	- No findings about specific cardiac changes or the direct impact of uremic toxins on cardiac function.

	HD compared to children on non-anuric HD.<br>- The %PB of HA, IAA, and IS were significantly reduced in children on anuric HD compared to children on non-anuric HD.<br>- β2M, pCG, HA, IAA, IS, and CMPF levels showed significant negative associations with residual urine volume but not UA or pCS levels.<br>- %PB was significantly correlated with residual urine volume for all protein-bound uremic toxins included.	

Abbreviations: ADMA: asymmetric dimethylarginine; Aix: augmentation index; β2M: Beta-2 microglobulin; cIMT: carotid intima-media thickness; CfD: complement factor D, CKD: chronic kidney disease; CMPF: 3-carboxy-4-methyl-5-propyl-2-furanpropanoic acid; CRS: cardiorenal syndrome; CV: cardiovascular; CVD: cardiovascular disease; eGFR: estimated glomerular filtration rate; ESKD: end-stage kidney disease; FAS: full age spectrum; HA: hippuric acid; HD: hemodialysis; IAA: indole acetic acid; IS: indoxyl sulfate; LVMI: left ventricular mass index; pCG: p-cresyl glucuronide; pCS: p-cresyl sulfate; PD: peritoneal dialysis; %PB: percentage of protein binding; PWV: pulse wave velocity; RFK: residual kidney function; SDMA: symmetric dimethylarginine; UA: uric acid.

Table S2. A summary of changes in uremic toxin concentrations in plasma/serum and cardiac parameter measurements in eligible articles.

Study	Uremic Toxins						Cardiac parameter measurements
	Sample Type	Assay	Timing of Collection	Small Water-soluble Uremic Toxin Concentrations	Middle Molecule Uremic Toxins Concentrations	Protein-bound Uremic Toxin Concentrations	
Crespo-Salgado et al., 2016 [19]	Serum	- HPLC	- Blood was collected immediately prior to the midweek dialysis session for HD patients.	Not applicable	Not applicable	**- IS**<br>**- pCS**	
Holle et al., 2019 [20]	Serum	- Not specified	- Blood and urine samples were collected every 6 months as part of routine visits; specific timing related to dialysis is not mentioned.	Not applicable	Not applicable	**- IS (µmol/L):**<br>- Total cohort: 25.3 ± 86.0; 5.3 (8.7)<br>- CKD stage 3a: 4.2 ± 11.7; 1.4 (1.5)<br>- CKD stage 3b: 16.5 ± 62.5; 3.4 (3.4)<br>- CKD stage 4: 30.7 ± 99.5; 7.3 (8.6)<br>- CKD stage 5: 43.9 ± 101; 15.6 (11.4)<br>**- pCS (µmol/L):**<br>- Total cohort: 21.0 ± 17.6; 17.0 (21.6)<br>- CKD stage 3a: 6.7 ± 05.9; 5.8 (7.5)<br>- CKD stage 3b: 13.9 ± 11.3; 10.4 (15.1)<br>- CKD stage 4: 24.5 ± 17.6; 20.9 (22.1)<br>- CKD stage 5: 38.8 ± 24.2; 35.1 (29.9)	**- cIMT SDS:**<br>- Total cohort: 1.62 ± 1.47<br>- CKD stage 3a: 1.83 ± 1.44<br>- CKD stage 3b: 1.47 ± 1.41<br>- CKD stage 4: 1.65 ± 1.46<br>- CKD stage 5: 1.89 ± 1.79<br>**- PWV SDS:**<br>- Total cohort: 0.34 ± 1.72<br>- CKD stage 3a: 0.36 ± 1.60<br>- CKD stage 3b: 0.25 ± 1.55<br>- CKD stage 4: 0.33 ± 1.82<br>- CKD stage 5: 0.86 ± 1.67<br>**- LVMI ($g/m^{2.16}$):**<br>- Total cohort: 41.4 ± 13.3<br>- CKD stage 3a: 35.8 ± 10.9<br>- CKD stage 3b: 40.2 ± 13.3<br>- CKD stage 4: 42.2 ± 12.2<br>- CKD stage 5: 45.0 ± 19.0
Holle et al., 2020 [21]	Serum	- HPLC	- Blood samples were collected as part of	Not applicable	Not applicable	**- IS (µmol/L):**<br>- Total cohort: 25.5 ± 86.4; 5.3 (8.7)<br>- CKD stage 3a: 4.0 ± 11.7; 1.4 (1.6)<br>- CKD stage 3b: 17.8 ± 65.6; 3.45 (3.70)	Not applicable

			routine visits; specific timing related to dialysis is not mentioned.			- CKD stage 4: 31.4 ± 105; 6.25 (7.30)<br>- CKD stage 5: 30.8 ± 78.3; 13.0 (11.0)<br>**- pCS (µmol/L):**<br>- Total cohort: 21.1 ± 18; 17.2 (22.0)<br>- CKD stage 3a: 6.99 ± 6.00; 6.2 (7.0)<br>- CKD stage 3b: 13.5 ± 11.2; 9.8 (15.2)<br>- CKD stage 4: 22.4 ± 16.1; 19.7 (19.7)<br>- CKD stage 5: 36.7 ± 22.0; 34.1 (30.0)	
Özdemir et al., 2015 [22]	Not specified	- Not specified	- Blood samples were collected 30 minutes before and 2 hours after dialysis.	**- Urea (mmol/L):**<br>- With CVD: 0.65 ± 0.13<br>- Without CVD: 0.66 ± 0.20<br>**- Creatinine (mg/dL):**<br>- With CVD: 0.56 ± 0.10<br>- Without CVD: 0.51 ± 0.26<br>**- Sodium [unspecified units]:**<br>- With CVD: 0.01 ± 0.09<br>- Without CVD: 0.01 ± 0.01<br>**- Potassium (mmol/L):**<br>- With CVD: 0.33 ± 0.16<br>- Without CVD: 0.21 ± 0.07<br>**- Phosphor (mg/dL):**<br>- With CVD: 0.56 ± 0.23<br>- Without CVD: 0.41 ± 0.09<br>**- Uric acid (mg/dL):**<br>- With CVD: 0.67 ± 0.13<br>- Without CVD: 0.59 ± 0.23<br>**- Glucose (mg/dL):**<br>- With CVD: 99 ± 14<br>- Without CVD: 96 ± 13	**- Parathormone (pg/mL):**<br>- With CVD: 0.27 ± 0.19<br>- Without CVD: 0.27 ± 0.14<br>**- Vitamin B12 (pg/mL):**<br>- With CVD: 0.12 ± 0.09<br>- Without CVD: 0.15 ± 0.08<br>**- β2M (mg/L):**<br>- With CVD: 0.04 ± 0.03<br>- Without CVD: 0.20 ± 0.07	**- Serum amyloid A (µg/mL):**<br>- With CVD: 0.09 ± 0.01<br>- Without CVD: 0.19 ± 0.08<br>**- Homocysteine (µmol/L):**<br>- With CVD: 0.12 ± 0.09<br>- Without CVD: 0.35 ± 0.03	**- AIx (%):**<br>- HD: 14.3 ± 2.1<br>- PD: 10.6 ± 1.3<br>- Control: 7.36 ± 3.59<br>**- PWV (m/s):**<br>- HD: 5.9 ± 0.9<br>- PD: 5.25 ± 0.6<br>- Control: 5.12 ± 0.85<br>**- cIMT (mm):**<br>- HD: 0.51 ± 0.11<br>- PD: 0.47 ± 0.09<br>- Control: 0.35 ± 0.12<br>**- LVMI (g/m²):**<br>- HD: 40.8 ± 2.5<br>- PD: 37.6 ± 4.3<br>- Control: 27.8 ± 1.55
Snauwaert et al., 2018 [23]*	Plasma	- HPLC<br>- ELISAs	- Blood samples were drawn during routine	**- Creatinine (z-score):**<br>- Overall: 8.9 [4.1; 21.7]<br>- CKD stages 1-2: 2.8 [1.3; 5.4]<br>- CKD stage 3: 8.9 [7.2; 12.1]<br>- CKD stage 4: 24.4 [20.5; 31.5]	**- CfD (z-score):**<br>- Overall: 9.5 [3.9; 21.2]<br>- CKD stages 1-2: 2.8 [1.8; 6.2]<br>- CKD stage 3: 9.5 [5.7; 11.9]<br>- CKD stage 4: 22.8 [16.2; 28.4]	**- pCG (z-score):**<br>- Overall: 0.5 [−0.8; 3.3]<br>- CKD stages 1-2: 0.1 [−1.0; 1.3]<br>- CKD stage 3: −0.5 [−1.0; 1.0]<br>- CKD stage 4: 3.8 [1.8; 9.8]	Not applicable

			ambulatory visits.	- CKD stage 5: 47.9 [41.1; 67.2]<br>**- SDMA (z-score):**<br>- Overall: 11.1 [5.2; 20.7]<br>- CKD stages 1-2: 4.8 [3.5; 7.0]<br>- CKD stage 3: 10.7 [6.5; 13.6]<br>- CKD stage 4: 25.7 [18.3; 29.6]<br>- CKD stage 5: 33.2 [23.4; 50.1]<br>**- ADMA (z-score):**<br>- Overall: −0.2 [−0.7; 1.2]<br>- CKD stages 1-2: −0.3 [−0.9; 1.1]<br>- CKD stage 3: −0.5 [−0.9; 0.7]<br>- CKD stage 4: 1.6 [−0.3; 2.3]<br>- CKD stage 5: 2.6 [−0.1; 3.7]	- CKD stage 5: 36.1 [28.3; 42.0]<br>**- β2M (z-score):**<br>- Overall: 8.6 [4.7; 18.1]<br>- CKD stages 1-2: 4.5 [2.4; 5.9]<br>- CKD stage 3: 9.5 [5.3; 12.9]<br>- CKD stage 4: 18.7 [16.7; 23.8]<br>- CKD stage 5: 48.1 [32.3; 65.1)]	- CKD stage 5: 6.6 [1.0; 49.2]<br>**- HA (z-score):**<br>- Overall: 1.7 [−0.1; 5.3]<br>- CKD stages 1-2: 0.3 [−1.0; 1.0]<br>- CKD stage 3: 2.0 [0.0; 4.6]<br>- CKD stage 4: 2.0 [0.3; 13.7]<br>- CKD stage 5: 20.2 [14.3; 21.6]<br>**- IAA (z-score):**<br>- Overall: 1.7 [0.3; 3.1]<br>- CKD stages 1-2: 0.4 [−0.6; 2.2]<br>- CKD stage 3: 1.7 [0.4; 2.7]<br>- CKD stage 4: 1.4 [0.6; 2.8]<br>- CKD stage 5: 5.2 [3.5; 16.9]<br>**- IS (z-score):**<br>- Overall: 5.0 [1.7; 16.3]<br>- CKD stages 1-2: 1.9 [0.0; 4.6]<br>- CKD stage 3: 2.4 [1.7; 7.6]<br>- CKD stage 4: 17.8 [13.5; 24.4]<br>- CKD stage 5: 32.2 [21.1; 52.9]<br>**- pCS (z-score):**<br>- Overall: 2.7 [0.5; 5.5]<br>- CKD stages 1-2: 0.7 [−0.4; 3.2]<br>- CKD stage 3: 2.1 [0.5; 3.8]<br>- CKD stage 4: 7.5 [4.7; 10.0]<br>- CKD stage 5: 7.8 [1.9; 19.8]<br>**- CMPF (z-score):**<br>- Overall: 2.1 [0.3; 5.3]<br>- CKD stages 1-2: 1.6 [0.0; 3.7]<br>- CKD stage 3: 2.9 [0.2; 5.3]<br>- CKD stage 4: 2.0 [0.9; 3.6]<br>- CKD stage 5: 5.7 [4.0; 13.6]	
Snauwaert et al., 2019 [24]	Plasma	- UPLC<br>- ELISAs	- Blood samples were collected	**- Uric acid (mg/dL)**<br>- CKD stages 4-5: 7.36 [6.55; 8.93]	**- β2M (µg/mL)**<br>- CKD stages 4-5: 9.92 [7.72; 15.4]	**- pCG (mg/dL)**<br>- CKD stages 4-5: 0.02 [0.01; 0.06]<br>- On HD: 0.15 [0.03; 0.33]	Not applicable

	during routine ambulatory visits in the non-dialysis CKD group and before a midweek HD session in the HD groups.	- On HD: 7.02 [5.80; 8.06]	- On HD: 30.7 [4.4; 39.3]	**- HA (mg/dL)**<br>- CKD stages 4-5: 0.39 [0.13; 0.64]<br>- On HD: 1.89 [0.72; 3.43]<br>**- IAA (mg/dL)**<br>- CKD stages 4-5: 0.06 [0.04; 0.08]<br>- On HD: 0.18 [0.12; 0.27]<br>**- IS (mg/dL)**<br>- CKD stages 4-5: 0.56 [0.44; 0.73]<br>- On HD: 2.04 [1.37; 2.70]<br>**- pCS (mg/dL)**<br>- CKD stages 4-5: 1.67 [0.93; 2.31]<br>- On HD: 2.35 [1.03; 3.27]<br>**- CMPF (mg/dL)**<br>- CKD stages 4-5: 0.05 [0.02; 0.21]<br>- On HD: 0.12 [0.03; 0.33]

*The concentrations of uremic toxins for each participant were presented as z-scores, compared to the reference values from a control population in Snauwaert et al. [23]. These comparisons were adjusted for different age categories as needed.

Data are mean ± SD, median (IQR), median [25th; 75th percentiles], or number (percentage) as appropriate.

Abbreviations: ADMA: asymmetric dimethylarginine; AIx: augmentation index; β2M: beta-2-microglobulin; cIMT: carotid intima-media thickness; CfD; complement factor D; CKD: chronic kidney disease; CMPF: 3-carboxy-4-methyl-5-propyl-furanpropionic acid; CVD: cardiovascular disease; ELISA: enzyme-linked immunosorbent assay; HA: hippuric acid; HD: hemodialysis; HPLC: high-performance liquid chromatography; IAA: indole acetic acid, IS: indoxyl sulfate; LVMI: left ventricular mass index; pCG: p-cresyl glucuronide; pCS: p-cresyl sulfate, PD: peritoneal dialysis; PWV: pulse wave velocity; SDMA: symmetric dimethylarginine; SDS: standard deviation score; UPLC: ultra-performance liquid chromatography.
